# The kallikrein-Kinin system modulates the progression of colorectal liver metastases in a mouse model

**DOI:** 10.1186/s12885-018-4260-6

**Published:** 2018-04-04

**Authors:** Patricia Luiza Nunes da Costa, David Wynne, Theodora Fifis, Linh Nguyen, Marcos Perini, Christopher Christophi

**Affiliations:** 1 Department of Surgery, University of Melbourne, Austin Health, Lance Townsend Building Level 8, Studley Rd, Heidelberg, VIC 3084 Australia; 2 Laboratório de Oncologia Experimental, Faculdade de Medicina da Universidade de São Paulo and Instituto do Câncer do Estado de São Paulo, São Paulo, Brazil

**Keywords:** Colorectal liver metastases, Kallikrein-Kinin system, Bradykinin receptor, Kinin or bradykinin

## Abstract

**Background:**

The Kallikrein-Kinin System (KKS) has been found to play a role in tumor progression in several cancers. The KKS metabolic cascade depends on signalling through two cross talking receptors; bradykinin receptor 1 (B1R) and bradykinin receptor 2 (B2R). Activation of the Kinin receptor is responsible for multiple pathophysiologic functions including increase of vascular permeability and induction of host inflammatory responses that exert diverse effects on tumor growth.

**Methods:**

B1R and B2R expression on mouse and human CRC cell lines was investigated. Changes in tumor growth and progression was assessed in male CBA mice bearing colorectal liver metastases (CRLM) following treatment with B1R or B2R blockers. In vitro cultures of human SW-480 and mouse colorectal cancer (MoCR) cell lines were examined for changes in their proliferation and migration properties following treatment with B1R or B2R blockers.

**Results:**

Both colorectal cancer cell lines tested strongly positive for B1R and B2R expression. Inhibition of both receptors retarded tumor growth but only B1R blockade significantly reduced tumor load and increased tumor apoptosis. Blockade of either receptor reduced tumor vascularization in vivo and significantly inhibited proliferation and migration of colorectal cancer cells in vitro.

**Conclusion:**

Taken together, the present study demonstrated that kinin receptor blockade inhibited tumor growth and reduced its invading properties suggesting that KKS manipulation could be a novel target in colorectal cancer therapy.

**Electronic supplementary material:**

The online version of this article (10.1186/s12885-018-4260-6) contains supplementary material, which is available to authorized users.

## Background

Colorectal cancer (CRC) is the third most common cancer worldwide [1]. The majority of CRC related deaths are associated with liver metastasis [2, 3]. Surgical removal of colorectal liver metastases (CRLM) supported by systemic chemotherapy provides the best possibility for cure in a percentage of patients and even among these, 30% - 60% will develop tumor recurrence in the liver or in other organs [4, 5]. Recent studies have demonstrated involvement of the renin angiotensin system (RAS) in cancer progression, including CRLM [6, 7]. There is also evidence of crosstalk between the Kallikrein Kinin System KKS and RAS pathways. The angiotensin-converting enzyme (ACE) catalyses both the production of angiotensin II and the degradation of bradykinin, suggesting a cross-regulation between the two systems [8], however the effects of the Kallikrein Kinin System (KKS) on CRLM have not been as well studied.

The role of kinin receptors in cancer has been investigated [9–11]. Many cancer tissues display higher expression of B1R and/or B2R compared to their normal counterparts [9]; ‘kinin’ in humans and most mammal contexts refers to bradykinin (BK). Signalling by BK through either B1R, B2R or both can promote angiogenesis in different experimental models by promoting vascular cell proliferation and survival, and by increasing vascular permeability [12, 13]. Selective antagonists of B1R or B2R have shown anti-proliferative, anti-inflammatory, anti-angiogenic and anti-migratory properties [9, 12, 14]. Kinin receptors have been reported in mouse and human colon carcinoma cell lines [15, 16]. BK treatment of the CRC cell line SW-480 results in mitogenic activation [16]. Zelawski et al. found a higher B1R expression in human tubular adenomas, a benign tumor that can become colonic carcinoma, suggesting that kinins may contribute to cellular transformation [17].

While the literature support the role of the Kallikrein Kinin System (KKS) signalling in tumor aggressiveness and progression, additional studies are needed in relevant animal models that closely represent the clinical tumor progression. We have developed and characterised a mouse model of CRLM in a fully immunocompetent host which we used in the present study to investigate the effects of kinin receptor inhibition on the CRLM tumors. Additionally we investigated the direct effects of KKS inhibitors on cultured CRC cell proliferation and migration.

## Methods

### Animals

CBA strain mice (Laboratory Animal services, University of Adelaide, South Australia) were used. Mice were kept in standard cages (2–5 animals per cage) in rooms with 12 h light/dark cycle, constant temperature and humidity. Food and water were provided ad libitum. Environmental enrichment was provided in the form of dry tissue ribbons and cardboard tunnels. All animal experiments were approved by the Austin Health Animal Ethics Committee.

#### Mouse model

Liver metastases were established by intrasplenic injection. as described previously [6, 18] Briefly, 2.5 × 10^4^ MoCR cells were injected into the spleen of 6 to 8 week old male CBA mice (body weight 24–28 g), under anaesthesia (isoflurane) following an abdominal incision to exteriorize the spleen. Mice were given long-acting analgesic (carprofen, 5 mg/kg) just before surgery, hydrating jelly post surgery and were monitored closely for the next 24 h. In this model tumors are well established by day 21 post-induction, when liver samples were collected and fixed in fresh 10% formalin.

#### Drugs/agents and treatments for the in vivo studies

Three groups of six animals were used, one control and two experimental groups. Animals were assigned randomly to each group. The B1R inhibitor (SSR240612) was given at 15 mg/kg/day. The B2R inhibitor (FR173657) was given at15 mg/kg twice daily (early morning and late afternoon), totalling 30 mg/kg/day. Both drugs were dissolved prior to administration in 10% DMSO diluted in distilled H_2_O. Control animals were administered 10% DMSO in distilled H_2_O. Each treatment was administered via oral gavage (volume of 0.25 ml) starting from day 1 post tumor induction.

#### Stereological assessment of tumor growth

Tumor volume and burden were assessed using quantitative stereology as described previously [19]. Briefly the fixed tissues were sliced into 1.5 mm sections and imaged using a Lumenera Infinity4 digital CCD camera. The imaging software Image-ProPlus 6.0 was used to collect the data. The researcher collecting the data was blinded in regards to the group origin of the tissue.

#### Immunohistochemistry

Immunohistochemistry and data collection were performed as described previously [20] Changes in tumor angiogenesis and apoptosis were determined by immunostaining formalin fixed liver tissues with anti-CD34 (rat anti-mouse monoclonal, Serotec MCA 18256 at 1 μg/ml and anti-Caspase 3 (rabbit polyclonal, R&D system AF835 at 2 μg/ml) antibodies, respectively. Antigen retrieval was performed by heat in citrate buffer.

#### Human and mouse cell lines

SW480 (ATCC® CCL-228™) cell line is a human colorectal cancer cell line, derived from grade 3–4 colon adenocarcinoma and grown adherent in cell culture. SW480 cells were cultured in DMEM/10% FBS at 37 °C and 5%CO_2_/95% air. The mouse MoCR cell line used in the current studies was originally derived in our laboratory from a dimethyl hydrazine-induced CBA mouse colon carcinoma as described previously [18] MoCR cells were cultured in RPMI/10% FBS at 37°Cand 5%CO_2_/95% air. Both cell lines were mycoplasma free upon testing. Culture passages 4–15 were used for the experiments described in these studies.

#### Drugs/agents and treatments for the in vitro studies

SSR240612 (Sanofi Aventis) was used at 10 μM to block B1R and FR173657 (Astellas Pharma) at 10 μM to block B2R. The agonist desArg9-Bradykinin (DABK) (Sigma-Aldrich) at 0.1 μM was used to activate B1R. Bradykinin fragment 1–8 (BK) (Sigma-Aldrich) at 0.1 μM was used to activate B2R.

#### Proliferation assay

Cell proliferation was determined by counting the incorporation of [H3]-thymidine. Cells were seeded at 6 × 10^4^ cells/well in RPMI/10%FBS (MoCR cells) or DMEM/10%FBS (SW480) in 96-well plates and allowed to adhere overnight before being starved in 0% FBS RPMI or DMEM for 18 h. Following starving, fresh RPMI/DMEM and 10 μCi/ml 3H-thymidine, +/− agonists (DABK or BK) and/or antagonists (SSR240612 or FR173657) were added and cultured for 48 h. The cell cultures were then collected using the NUNC cell harvester. The 3H-thymidine incorporation into the dividing cells was measured using a β-counter (Packard, Meriden, CT).

#### Cell migration/invasion assay

A modified Boyden Chamber assay [21] was used to investigate cell migration and invasion. Briefly membranes (8-μm pore size, BD353097, Becton Dickinson, NJ, USA) were coated with 30 μg/30 μl fibronectin (BD3534009, Becton Dickinson, USA) on their lower surface and placed into the wells of a 24-well plate containing 600 μl/well serum-free RPMI and 0.1% BSA. Tumor cells (2–5 × 10^4^ cells/100 μl) were added to the upper chamber in the presence or absence of agonists (DABK or BK) and/or antagonists (SSR240612 or FR173657). The plates were placed in a humidifying incubator for 24 h at 37 °C and 5% CO_2_. The cells on the upper surface of the membranes were removed and the cells on the lower surface were fixed, stained with Quick-Dip (Fronine, Sydney, Australia) and counted using a NIKON Coolscope (Coherent Scientific, Adelaide, Australia).

#### Statistical analysis

Data was analysed using the statistical software package SPSS (SPSS Version 17.0; IBM Co., Armonk, NY, USA). Quantitative data are presented as mean values ± S.E.M. for each group. Parametric data was analysed using ANOVA with post hoc comparison (Tukey method) for parametric data and Mann-Whitney U test for non-parametric data. A *p* value ≤0.05 was considered statistically significant.

## Results

### Mouse and human CRC cell lines express B1R and B2R.

We first wanted to establish whether our CRC cell lines (MoCR and SW480) express B1R and/or B2R. Immunohistochemical staining for these receptors confirmed their expression (Additional file 1), supporting previous published studies that CRC cells express bradykinin receptors [22].

### B1R blockade reduced tumor burden in a mouse model.

**Fig. 1:**
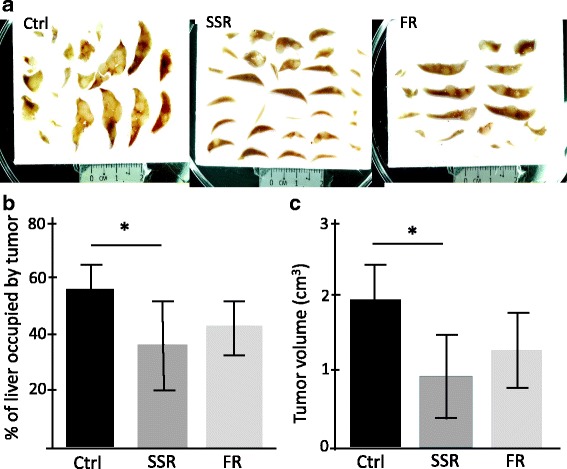
The effect of kinin receptor inhibition on CRLM tumor growth in CBA mouse model. CBA mice, 6 per group, underwent tumor induction surgery and were treated with SSR240612 (SSR) (15 mg/kg/day), FR173657 (FR), (30 mg/kg/day) or vehicle solution (Ctrl) daily via oral gavage starting at day 1 post tumor induction. Livers were collected on day 21 and were fixed in formalin. **a** represtative images of liver sections used to determine percentage tumor occupancy and total tumor volume were determined by stereological analysis using Image ProPlus 6. **b** Percentage tumor occupancy of the liver and **c** total tumor volume were significantly reduced following SSR240612 treatment Results are presented as mean±SD, (*p < 0.05 compared to control)

To investigate whether modulation of signalling through the B1R or the B2R affects tumor progression, B1R (SSR240612) or B2R (FR173657) blockers were given to six mice/group harbouring tumor metastases. Quantitative stereology analysis of tumor growth at day 21 post tumor induction demonstrated a decrease in tumor load following both treatments, however this did not reach significance for B2R blocker (P˂0.05 for the B1R blocker and *P* = 0.16 for the B2R blocker) (Fig. 1A). Similarly there was tumor volume reduction compared to control (P˂0.05 for the B1R blocker and *P* = 0.12 for the B2R blocker) (Fig. 1B).

**Fig. 2:**
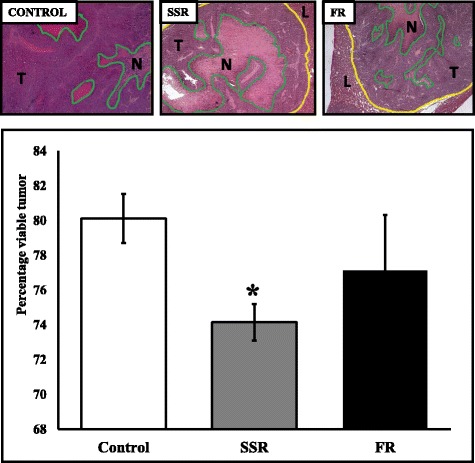
Effect of kinin receptor inhibition on viable tumor growth in CBA mice induced with CRLM. CBA mice, 6 per group, underwent tumor induction surgery and were treated with SSR240612 (SSR), (15 mg/kg/day), FR173657 (FR), (30 mg/kg/day) or vehicle solution (CONTROL) daily starting at day 1 post tumor induction via oral gavage. Livers were collected on day 21post tumor induction. Percentage of viable tumor was quantitated by Haematoxylin and Eosin staining (necrotic regions display absence of cell membranes and pycnotic nuclei if present). 10 to 20 images at 5× magnification of stained tumor from each mouse were taken in a pre-determined systematic fashion. Yellow lines show tumor area, T = live tumor area, N = necrotic tumor enclosed within green lines. SSR treatment significantly decreased the percentage of viable tumor load in animals (*P = 0.016). The reduction in viable tumor load was not observed following FR treatment compared to control (*P* = 0.88). Results are presented as group mean value ± SD

Further analysis focusing on the percentage of viable tumor following SSR and FR treatments revealed a significantly lower percentage of viable tumor load in animals treated with the B1R blocker SSR (8% less viable tumor load, *P = 0.016) (Fig. 2). No reduction in viable tumor load was observed following FR treatment compared to control (*P* = 0.88) (Fig. 2).

#### B1R blockade increased tumor apoptosis in vivo

**Fig. 3:**
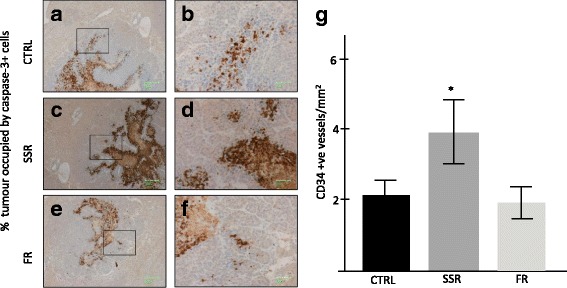
Effect of kinin receptor inhibition on tumor cell apoptosis in CBA mice induced with CRLM. (CTRL=control, SSR=SSR240612, FR=FR173657). Apoptotic tumor cells in CRLM tumors were visualised by immunostaining for caspase-3 (brown staining). **a**, **c** &**e** magnification 5×, **a**, **d** &**f** (corresponding insets) magnification 20×. 40 to 100 images at 20× magnification of stained tumor from each mouse were taken in a pre-determined systematic fashion. Caspase-3 positive cells were counted as a percentage of tumor area using Image ProPlus 6. SSR240612 treatment significantly increased the number of tumor cells undergoing apoptosis. Results are presented as group mean value ± SD, (*p < 0.05 compared to control), *n* = 6

We further investigated the effect of kinin receptor blockade on tumor cell apoptosis. Immunohistochemistry staining demonstrated significant increase in tumor cells undergoing apoptosis following SSR treatment. Tumors treated with SSR had 86% more apoptosis compared to controls (*p < 0.05); FR treatment did not increase apoptosis compared to controls (Fig. 3).

#### Kinin receptor blockade inhibited angiogenesis

**Fig. 4:**
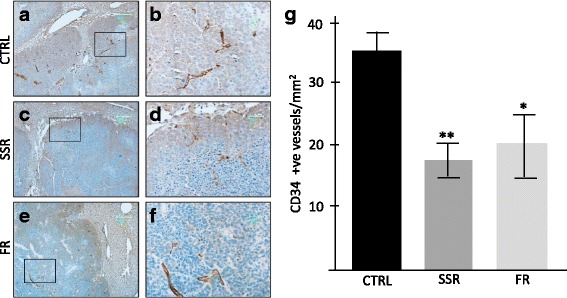
Effect of kinin receptor inhibition on tumor angiogenesis in CBA mice induced with CRLM. Tumor blood vessels were visualised by immunostaining for CD34 endothelial cell marker (brown staining). **a**,**c**&**e** magnification 5×, **a**,**d**&**f** (corresponding insets) magnification 20×, 0 to 100 images at 20× magnification of stained tumor from each mouse were taken in a pre-determined systematic fashion. CD34 positive blood vessels were counted using Image ProPlus 6. SSR240612 (SSR) and FR173657 (FR) treatment significantly reduced tumor angiogenesis compared to control (CTRL) shown in **g**. Results are presented as group mean value ± SD, (n = 6) (*p < 0.001; **p < 0.0001 compared to control)

Previous studies have suggested an important role of KKS in promoting angiogenesis, increasing vascular permeability and expression of VEGF in solid tumors. To investigate if this is also a mechanism of tumor modulation in our model we examined the tumors for changes in vessel density following treatment with B1R and B2R receptor blockers. The results demonstrate both SSR and FR treatment resulted in significant reduction in angiogenesis compared to controls (48%, **p < 0.0001; 39%, *p < 0.001, respectively) (Fig. 4).

### Bradykinin receptor blockers inhibited in vitro tumor cell proliferation

Having established that blocking the B1R and B2R signalling slows CRC tumor progression and that CRC tumor cells express bradykinin receptors we wanted to know if receptor activation or blockade modulate tumor cell characteristics directly. The stimulation of the receptors on cell proliferation was also examined in MoCR cells by the addition of agonists 0.1 μM DABK (B1R) or BK (B2R). Exposure to 0.1 μM of DABK did not significantly change the proliferation of MoCR cells. In contrast 0.1 μM of BK increased MoCR proliferation by 30% at 48 h after treatment (*P < 0.05) (Additional file 2).

**Fig. 5:**
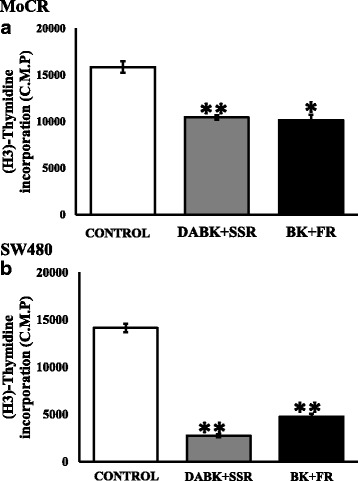
The effect of kinin receptor antagonists on MoCR and SW480 cell proliferation. **a** MoCR cells and **b** SW480 cells were cultured for 48 h in RPMI media in the presence of 0.10 μM of kinins (BK or DABK) and 10.0 μM of the B2R antagonist FR173657 (FR) or the B1R antagonist SSR240612 (SSR) in specific combinations. The control was RPMI media with no FBS, SSR240612 and FR173657 treatment significantly reduced tumor cell proliferation compared to control cells. Results are presented as replicate mean ± SD, 4 replicate per group (*p < 0.001; **p < 0.0001 compared with untreated control)

We then investigated the effects of the inhibitors on MoCR and SW480 cell cultures. The cultures were treated with the receptor blockers SSR240612 (B1R) or FR173657 (B2R) at 10 μM in the presence or absence of the respective agonists and their effect on cell proliferation was determined using proliferation assays as described. Significant reduction in proliferation was seen in both cell lines (Fig. 5) following SSR (**P < 0.0001 for both cell lines) and FR treatment (*p < 0.001, in MoCR and **p < 0.0001 in SW480). The reduction of proliferation was the same whether the agonists were present (Fig. 5) or absent. (Not shown).

### B1R and B2R blockade inhibited kinin mediated invasion in CRC

**Fig. 6:**
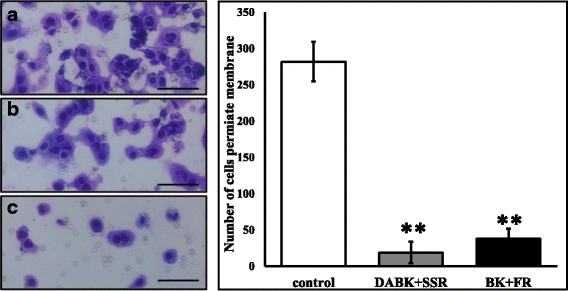
The effect of kinin receptor antagonism on MoCR cell invasion. MoCR cells were seeded into upper chambers with kinin/ kinin-receptor inhibitor combinations in lower chambers. The membrane on the lowers surface of the upper chamber was coated with fibronectin and allowed to set. **a** Non-treated cells, **b** Cells treated with 10.0 μM of SSR240612 (SSR) and 0.10 μM DABK, **c** Cells treated with 10.0 μM of FR173657 (FR) and 0.10 μM BK, [E] Number of cells that permeated the membrane. Cells were culture for 21 h in 0.01% BSA RPMI media. The control was 0.01% BSA RPMI media. SSR240612 and FR173657 treatment significantly reduced the number of invasive MoCR cells. Results are presented as replicate mean ± SD, 2 replicates per group (**p < 0.0001 compared to control). 40X magnification; Bar = 25 μm

Treatment with 0.1 and 1 μM BK promoted cell invasion in MoCR cells (Additional file 3). The effect of B1R and B2R blockade on tumor cell invasion in the presence of agonists was also tested. Invasion assays were performed following DABK+SSR or BK + FR treatment and quantified 21 h later. As shown in Fig. 6, addition of kinin receptor blockers resulted in over 90% reduction of invasion in MoCR cells. Treatment with agonists in the presence of inhibitors (DABK+SSR and BK + FR) did not restore invasion (p** < 0.0001) compared to untreated controls.

## Discussion

The KKS is a complex multifunctional signalling cascade and its role in cancer remains unclear. Early reports on the likely role of the KKS in cancer suggested enhanced vascular permeability and upregulation of nitric oxide synthase and prostaglandin [23, 24]. This led to the suggestion that KKS family members may be novel biomarkers for cancer [25–28]. More recent studies further support the role of the KKS in cancer development [7, 29] and a number of KKS generated proteins confirmed to exhibit pro-inflammatory properties [30]. Chronic inflammation is tightly associated with cancer development and progression [31].

In this study B1R or B2R blockade led to reduction in tumor progression compared to untreated controls, however this reduction did not reach significance for B2R blockade. Blockade of B1R resulted in significant viable tumor reduction and significant increase in apoptosis suggesting that the reduction in tumor progression is effected, at least in part, through tumor cell apoptosis. In contrast there was no reduction in the percentage of viable tumor or increase in apoptosis associated with B2R blockade. It has been shown that B1R and B2R can activate several intracellular signalling pathways including NF-κB [32, 33]. Indeed the p53 and NF-κB pathways are, arguably, the two major cellular stress response pathways leading to pro-apoptosis or anti-apoptosis pathways respectively. Webster et al. demonstrated that during injury or stress there is crosstalk between NF-κB and p53; they showed that these two transcription factors can modulate each other’s functions depending on their relative levels present. Following injury, the ability of NF-kB to supress p53 can determine the cell fate [34]. Although both B1R and B2R have been shown to induce NF-kB activation; the patterns of signalling are different in terms of duration and intensity [35]. It is possible that B1R activation of NF-kB leads to anti-apoptotic properties, therefore inhibition of B1R demonstrated a significant increase in tumor apoptosis. Another possible explanation for the reduced tumor burden following B1R blockade could be due to a reduction in chronic inflammation normally seen in cancer as a result of increased expression and signalling of the B1R. In contrast B2R is constitutively expressed and hence assumed to be mostly responsible for effecting normal physiological functions.

Our results show that blockade of either the B1R or the B2R resulted in significant reduction in tumor vascular density and this may be one of the mechanisms by which tumor progression is retarded. B1R and B2R activation have been implicated in angiogenesis and neovascularization in other studies. Kinins induce EC proliferation in cell cultures through the B1-cAMP pathway. B1R stimulation has been shown to induce neovascularization in the rabbit cornea [36], while B2R stimulation can activate the mitogen-activated protein kinase pathways (MAPK) and P13K/AKT, contributing to proliferation and angiogenesis [37–39]. Other studies have shown tumor suppression and angiogenic inhibition following treatment with B2R inhibition [40] or in kininogen knock out mice [12]. Moreover, Morbidelli et al. demonstrated the ability of BK to stimulate EC proliferation via activation of B1R or indirect activation of B2R, further supporting a role of kinin receptors in angiogenesis and tumor development [41].

In agreement with Wang et al. [22] we also detected B1R and B2R expression in both MoCR and SW480 colon cancer cells lines suggesting that receptor blockade may also have direct effects on the tumor cells. There are several studies which suggest that activation of B1R and B2R leads to stimulation of tumor cell proliferation and migration [37, 42, 43]. Unlike Barki-Harrington et al. who found increased proliferation following stimulation of B1R and B2R [42], our in vitro study failed to show increased proliferation with B1R agonist (desArg9-Bradykinin (DABK)), however, the involvement of B1R in proliferation cannot be completely ruled out, since treatment with a B1R antagonist (SSR240612) demonstrated significant reduction in tumor proliferation. McLean et al. found that the synthesis of DABK is significantly upregulated during inflammation [44]; it is possible that MoCR cells may have maximal DABK secretion therefore external addition of the DABK agonist does not further increase cell proliferation. Although DABK failed to increase tumor proliferation, our current study did find that BK treatment (B2R agonist) resulted in increased cell proliferation and the use of a B2R antagonist (FR173657) significantly decreased proliferation, further demonstrating that kinins do play a role in tumor proliferation. Further stimulation of kinin receptors was shown to increase tumor cell migration. Wang et al. reported that treatment with BK stimulated B2R and ERK1/2 leading to increased IL-6 production, ultimately increasing the invasiveness of colorectal cancer cells [22]., Ehrenfeld et al. found that activation of the B1R increased secretion of the metalloproteases (MMPs)-2 and − 9 by breast cancer cells [11]. Previous studies have shown that MMP2 activates integrin αvβ3 resulting in cellular invasion [45]; similarly MMP-9 has been shown to enhance metastatic capacity through activation of αvβ3 in breast cancer cell lines [46] and αvβ6 integrin activation has been shown to promote invasion of squamous cell carcinoma cells [45]. In agreement, using invasion assays, we observed significant decrease in tumor invasion following treatment with both B1R and B2R antagonists, further confirming a role of bradykinin receptors in CRLM migration.

## Conclusion

Here we show that kinin receptors are involved in MoCR tumor progression In an in vivo CRLM mouse model tumor angiogenesis is inhibited and tumor progression retarded by B1R and B2R blockade. In addition, B1R inhibition led to significant reduction in the percentage of live tumor, possibly due to the increased tumor apoptosis. In an in vitro setting B1R and B2R antagonists decreased both tumor proliferation and migration. Taken together the results indicate that KKS manipulation could be a novel therapeutic target for treatment of colorectal cancer.

## Additional files

Additional file 1: Immunocytochemistry for detection of B1R and B2R on the colorectal cancer cell lines MOCR and SW480. [A] MoCR cells stained with naive rabbit IgG, [B] MoCR cells stained with rabbit anti-B1R, [C] SW480 cells stained with naive rabbit IgG, [D] SW480 cells stained with rabbit anti-B1R, [E] MoCR cells stained with naive rabbit, [F] MoCR cells stained with rabbit anti-B2R, [G] SW480 cells stained with naive rabbit IgG, and [H] SW480 cells stained with rabbit anti-B2R. Images taken at 20× magnification. Bar = 50 μm. (PDF 178 kb)

Additional file 2: The proliferative effect on MoCR cells exposed to kinins. MoCR cells were cultured for 48 h in RPMI media treated with [A] BK (0.001, 0.01, 0.10 and 1.0 μM) and [B] DABK (0.001, 0.01, 0.10, 1.0 and 10.0 μM). The negative control was RMPI media contain no FBS; the positive control was RPMI media containing 5% FBS. Results are presented as mean ± SD, 4 replicates per group (*p < 0.05 compared to negative control). (PDF 157 kb)

Additional file 3: Effects of BK on MoCR cell migration/invasion. A Boyden chamber assay was used. Transwell inserts (8 μm pore size membrane) were coated with fibronectin. Cells were loaded into the upper chamber and BK was added to the lower chamber containing serum-free media. The number of invading cells was counted after staining the membrane with eosin/thiazine. Exposure of 0.1 and 1 μM BK increased MoCR cell migration in a dose dependent manner. Results are from three independent experiments in triplicate (average ± SEM), *p < 0.05. (PDF 204 kb)

## Abbreviations

CRLM: colorectal liver metastasis
KKS: Kallikrein-Kinin System
MoCR: Mouse colorectal cancer
