# Supplementary material for: The kallikrein-Kinin system modulates the progression of colorectal liver metastases in a mouse model
Source: BMC Cancer. 2018 Apr 4;18:382. doi: 10.1186/s12885-018-4260-6 (PMC5885419; doi:10.1186/s12885-018-4260-6)
Supplement: Supplementary file 1 — Immunocytochemistry for detection of B1R and B2R on the colorectal cancer cell lines MOCR and SW480. [A] MoCR cells stained with naive rabbit IgG, [B] MoCR cells stained with rabbit anti-B1R, [C] SW480 cells stained with naive rabbit IgG, [D] SW480 cells stained with rabbit anti-B1R, [E] MoCR cells stained with naive rabbit, [F] MoCR cells stained with rabbit anti-B2R, [G] SW480 cells stained with naive rabbit IgG, and [H] SW480 cells stained with rabbit anti-B2R. Images taken at 20× magnification. Bar = 50 μm. (PDF 178 kb) [file 12885_2018_4260_MOESM1_ESM.pdf]

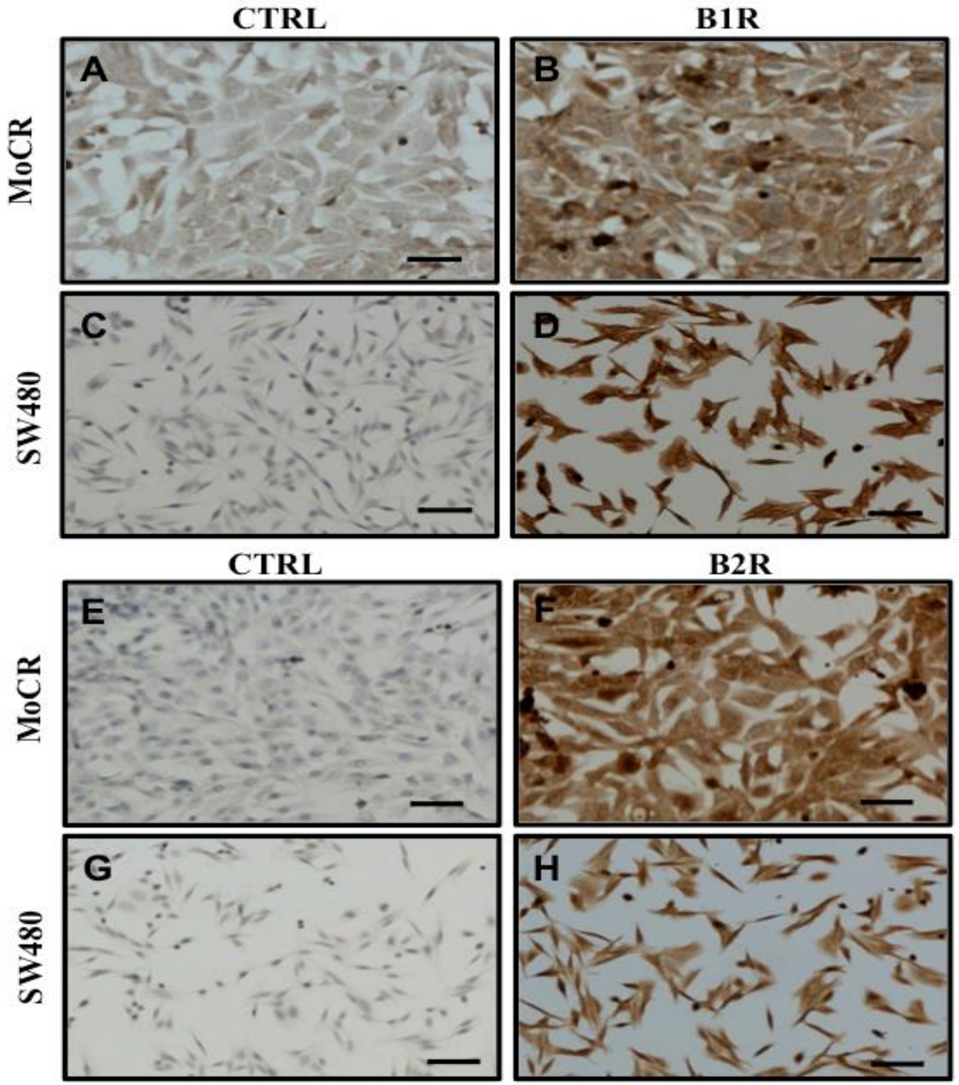

Additional File 1: Immunocytochemistry for detection of B1R and B2R on the colorectal cancer cell lines MOCR and SW480. [A] MoCR cells stained with naive rabbit IgG, [B] MoCR cells stained with rabbit anti-B1R, [C] SW480 cells stained with naive rabbit IgG, [D] SW480 cells stained with rabbit anti-B1R, [E] MoCR cells stained with naive rabbit, [F] MoCR cells stained with rabbit anti-B2R, [G] SW480 cells stained with naive rabbit IgG, and [H] SW480 cells stained with rabbit anti-B2R. Images taken at 20x magnification. Bar = 50µm.
