# Supplementary material for: The kallikrein-Kinin system modulates the progression of colorectal liver metastases in a mouse model
Source: BMC Cancer. 2018 Apr 4;18:382. doi: 10.1186/s12885-018-4260-6 (PMC5885419; doi:10.1186/s12885-018-4260-6)
Supplement: Supplementary file 2 — The proliferative effect on MoCR cells exposed to kinins. MoCR cells were cultured for 48 h in RPMI media treated with [A] BK (0.001, 0.01, 0.10 and 1.0 μM) and [B] DABK (0.001, 0.01, 0.10, 1.0 and 10.0 μM). The negative control was RMPI media contain no FBS; the positive control was RPMI media containing 5% FBS. Results are presented as mean ± SD, 4 replicates per group (*p < 0.05 compared to negative control). (PDF 157 kb) [file 12885_2018_4260_MOESM2_ESM.pdf]

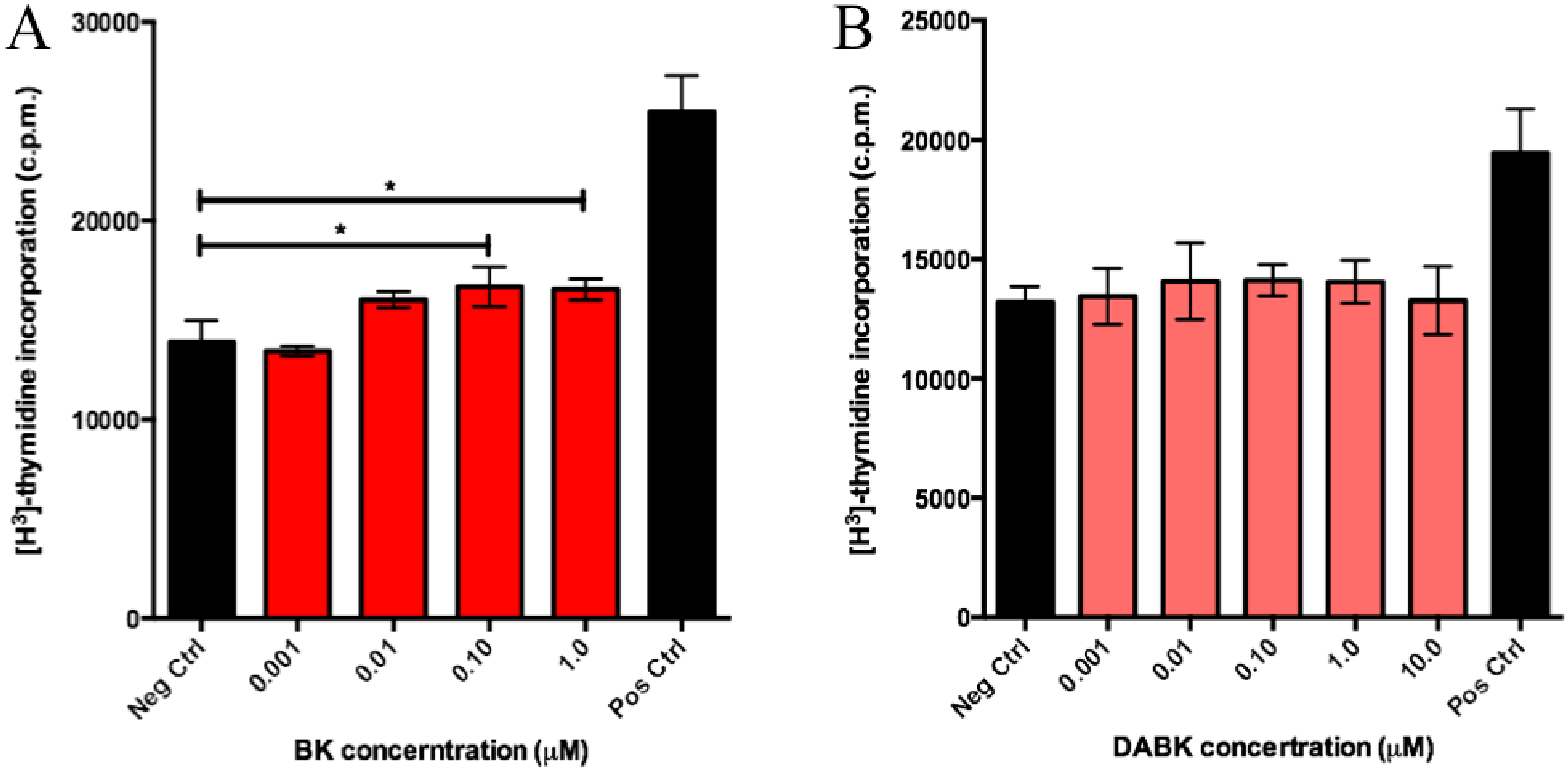


**Additional file 2. The proliferative effect on MoCR cells exposed to kinins.** MoCR cells were cultured for 48 hours in RPMI media treated with [A] BK (0.001, 0.01, 0.10 and 1.0 μM) and [B] DABK (0.001, 0.01, 0.10, 1.0 and 10.0 μM). The negative control was RMPI media contain no FBS; the positive control was RPMI media containing 5% FBS. Results are presented as mean ± SD, 4 replicates per group (*p<0.05 compared to negative control).
