# Supplementary material for: The kallikrein-Kinin system modulates the progression of colorectal liver metastases in a mouse model
Source: BMC Cancer. 2018 Apr 4;18:382. doi: 10.1186/s12885-018-4260-6 (PMC5885419; doi:10.1186/s12885-018-4260-6)
Supplement: Supplementary file 3 — Effects of BK on MoCR cell migration/invasion. A Boyden chamber assay was used. Transwell inserts (8 μm pore size membrane) were coated with fibronectin. Cells were loaded into the upper chamber and BK was added to the lower chamber containing serum-free media. The number of invading cells was counted after staining the membrane with eosin/thiazine. Exposure of 0.1 and 1 μM BK increased MoCR cell migration in a dose dependent manner. Results are from three independent experiments in triplicate (average ± SEM), *p < 0.05. (PDF 204 kb) [file 12885_2018_4260_MOESM3_ESM.pdf]

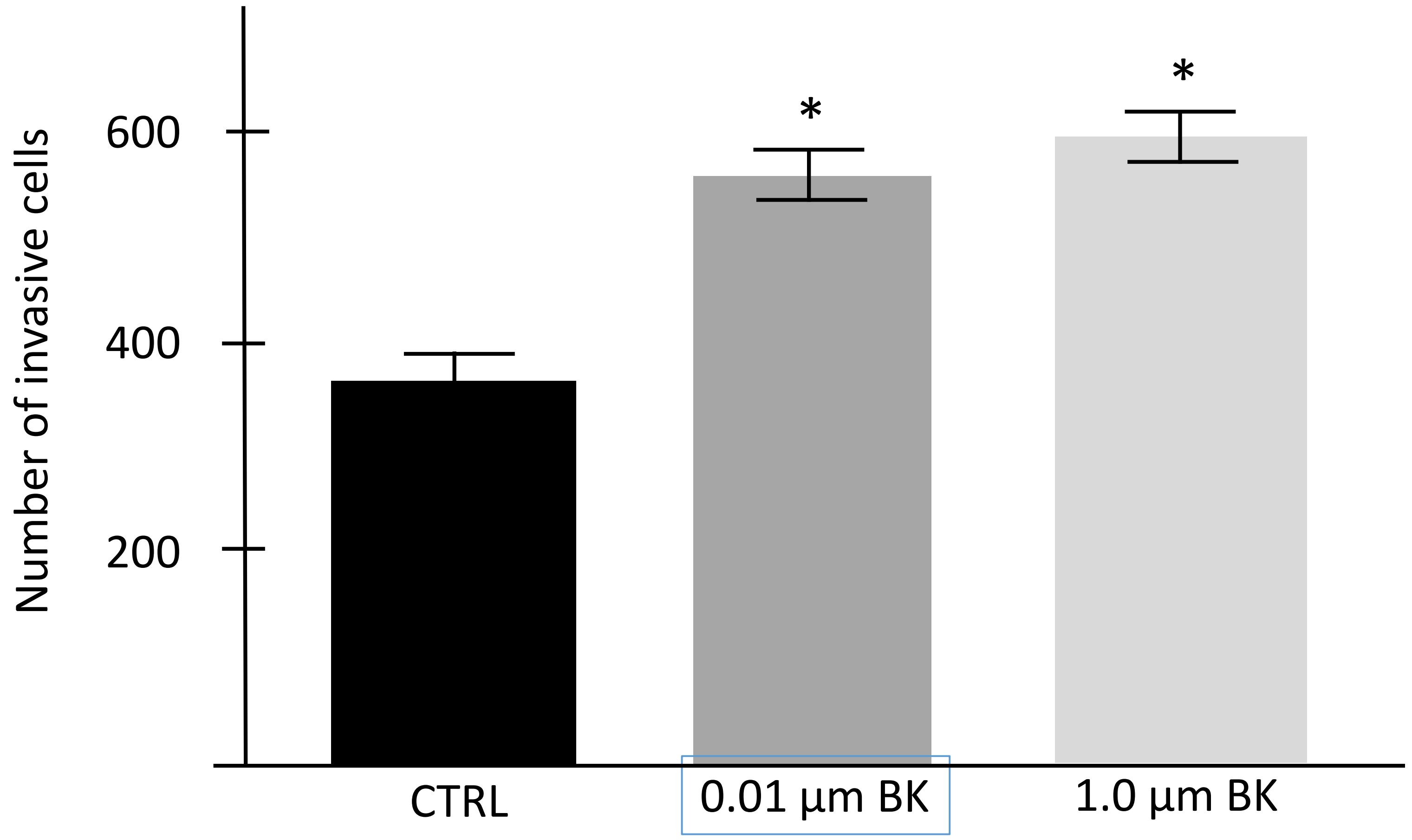

Additional File 3: Effects of BK on MoCR cell migration/invasion. A Boyden chamber assay was used. Transwell inserts (8 µm pore size membrane) were coated with fibronectin. Cells were loaded into the upper chamber and BK was added to the lower chamber containing serum-free media. The number of invading cells was counted after staining the membrane with eosin/thiazine. Exposure to 0.1 and 1µM BK increased MoCR cell migration in a dose dependent manner. Results are from three independent experiments in triplicate (average ± SEM), *$p<0.05$.
